# A New Method of Assessing Endometrial Compaction as an Indicator of Endometrial Receptivity for Predicting Reproductive Success

**DOI:** 10.3390/jcm14227923

**Published:** 2025-11-08

**Authors:** Robert Milewski, Magdalena Skowrońska, Agnieszka Kuczyńska, Andrei Lebedzko, Waldemar Kuczyński

**Affiliations:** 1 Department of Biostatistics and Medical Informatics, Medical University of Bialystok, 15-295 Bialystok, Poland; 2 Doctoral School, Medical University of Białystok, 15-276 Białystok, Poland; magdalena.skowronska@sd.umb.edu.pl; 3 Kriobank Infertility Treatment Center, 15-879 Bialystok, Poland

**Keywords:** endometrial compaction, endometrial receptivity, implantation prediction, assisted reproductive technologies

## Abstract

**Background/Objectives**: Accurate prediction of reproductive outcomes remains a key challenge in assisted reproductive technologies (ARTs). While embryo quality assessment has been extensively studied, endometrial receptivity has received less attention despite its critical role in implantation. Endometrial compaction (EC), i.e., the reduction in endometrial thickness between ovulation and embryo transfer, has been proposed as a potential predictor, but the current literature data is inconclusive. This study aimed to develop and validate a novel implantation predictor (IMP), based on extended assessment of endometrial shape and dynamics, which would be useful in determining reproductive success. **Methods:** The study analyzed data from 61 couples undergoing infertility treatment at the Kriobank Clinic (Białystok, Poland) between December 2021 and February 2025. Endometrial measurements were taken at the day following the ovulatory peak and on the day of embryo transfer. A set of normalized parameters describing endometrial dimensions was proposed and their changes over time measured. Based on the obtained data, a multivariable logistic regression model was constructed to create the IMP. **Results:** The proposed model demonstrated high predictive power for implantation, with an AUC of 0.839 (95% CI: 0.739–0.938). Statistically significant differences in IMP values were observed between the pregnancy and no-pregnancy groups (*p* < 0.0001). Quartile analysis showed that implantation rates increased from 6.25% in the lowest IMP range to 93.3% in the highest, confirming the model’s strong predictive power. In the study group, the model is capable of predicting a quarter of cases in which implantation will almost certainly occur and another quarter in which implantation will almost certainly not occur. **Conclusions:** This study introduces a novel predictor (IMP) of implantation based on an extensive assessment of endometrial compaction, which may be used in predicting reproductive success. The findings show the importance of considering endometrial receptivity in ART success. They also indicate that integrating IMP with existing approaches may substantially improve predicting reproductive success.

## 1. Introduction

Infertility is defined as a condition in which a couple is unable to achieve a clinical pregnancy after 12 months of regularly performing unprotected sexual intercourse [1]. This problem concerns women as well as men [2]. As its scale continues to grow and has reached a global dimension, there is still a pressing need for new, well-designed scientific studies in this field. According to estimates, infertility affects as many as 48 million couples worldwide [3], with the highest prevalence of infertility observed in East and South Asia, as well as in Eastern Europe [4]. Certain forecasts predict a further increase in the number of people struggling with infertility, as well as a growing need to improve the effectiveness of treatment [5], which indicates the importance of conducting further research in this area.

Many methods of infertility treatment exist, which are individually catered to the patient’s—or couple’s—needs, based on the diagnosed underlying cause [6]. The treatment protocol should include those methods that offer the greatest chances of achieving pregnancy and giving birth to a healthy child. Infertility treatment mainly relies on pharmacotherapy, surgical treatment, and assisted reproductive technologies (ARTs) [7]. In situations where causal treatment is not recommended or proves ineffective, the use of ART can increase the chances of a positive outcome, i.e., achieving pregnancy and giving birth to a child [7]. The last few decades have seen dynamic development in ART, which has enabled effective treatment of cases previously considered incurable [8].

Despite the aforementioned advancements, it is agreed that the effectiveness of ART could be higher. An important step in improving treatment effectiveness is the accurate prediction of reproductive outcomes. In this respect, determining the developmental potential of embryos would allow for a more precise selection of the embryo for transfer, thus increasing the chances of success. Additionally, a faster transfer—on days 2 or 3, instead of waiting until days 5 or 6 to assess blastocyst quality—would shorten the in vitro culture period and thus reduce treatment costs. Several models have been developed specifically for assessing the developmental potential of embryos in the IVF process. Some of them were morphokinetic models [9,10,11], which yielded predictors with an AUC of around 0.7 [12,13] for predicting biochemical pregnancy, clinical pregnancy, or live birth. A higher AUC of around 0.8 [14] was achieved by models that predicted development to the blastocyst stage.

Aspects involving the endometrium—its receptivity, readiness to accept the developing embryo, and ability to provide an optimal environment for its development—are significantly less studied. Despite this fact, it should be emphasized that researchers focusing on predicting treatment effectiveness based on embryo quality assessment, for example, through morphokinetic evaluation, often indicate that endometrial receptivity is a complementary area of research that needs to be studied in order to increase the accuracy of prediction of reproductive success. One of such directions proposed in the literature is the assessment of endometrial compaction (EC) [15,16]. The approach consists of evaluating changes in endometrial thickness between the day following the ovulatory peak and the day of embryo transfer as an indicator that the endometrium has compacted, i.e., it has become receptive and conducive to implantation—with the authors concluding that endometrial compaction significantly increases the chances of implantation [16,17,18,19,20]. Other findings, however, do not support this conclusion [21,22,23,24,25]. One possible explanation for the discrepancies is that a single measurement of endometrial thickness appears to lack sufficient precision—both due to the different techniques and the choice of measurement site, as well as the nature of processes occurring in the endometrium, which affect its shape, between the two time points mentioned above [26].

A key aspect of the published research that may be crucial in the context of using changes in endometrial thickness for predictive purposes is the fact that authors of previous studies do not in fact specify how endometrial thickness was measured. This omission makes it likely that differing methods, measurement sites, or other additional factors could have been used—potentially leading to varying results. This lack of both methodological clarity and, most probably, homogeneity may explain why certain studies showed significant findings while others did not. This particular inconsistency has led the authors of the present study towards the idea of proposing a more detailed assessment of endometrial shape—based on a greater number of parameters compared to a single, vaguely defined, “endometrial thickness” measurement—as well as an evaluation of changes occurring between the day following the ovulatory peak and the day of embryo transfer. In this context, it is worth mentioning that some studies indicate that 3D measurement of the endometrium may provide more precise results [27], owing to its ability to reflect more complex processes undergone by the endometrium. Based on the approach outlined above, the authors planned to develop a completely novel predictor of embryo implantation (IMP), based on the dynamics of endometrial changes. The term ‘dynamics’ as used in this article denotes changes in endometrial thickness occurring between the two aforementioned time points, assessed by measuring several parameters, which de facto describe the shape of the endometrium.

Hence, the aim of the present study is to assess the extent to which information related solely to endometrial receptivity—specifically, the dynamics of compaction—can predict reproductive success.

If high predictive accuracy of the approach is confirmed, further research will be conducted, based on the results of this study, aimed at integrating the developed model with morphokinetic data, describing embryo developmental potential, as well as the woman’s age, and other clinically significant variables. The aim would be to create a highly functional model with predictive power exceeding that of all currently available models used to predict the success of infertility treatment via IVF.

## 2. Materials and Methods

The study was conducted in 2025 using data obtained from couples treated for infertility at the Kriobank Clinic (Białystok, Poland) between December 2021 and February 2025. Data from 61 couples were analyzed, including 27 couples who achieved implantation and 34 who did not. Each woman underwent two ultrasound examinations of the uterine endometrium: one on the day following the ovulatory peak—detected based on urinary ovulation tests—and the other on the day of embryo transfer into the uterine cavity. Embryo transfer into the uterus was performed on the 7th day after the ovulatory peak. The following parameters were measured on each of the two ultrasound images:

− l1—uterine length, measured between the highest point of the uterine fundus and the cervical opening;
− w1—width at the widest point of the endometrial outline;
− l2—distance measured along the junction defined during the l1 measurement, limited to the segment between the highest point of the uterine fundus and the intersection with the w1 measurement;
− w2, w3, w4—subsequent widths of the endometrial outline, measured along segments parallel to the w1 measurement and equidistant from each other by l2, continuing towards the cervical opening.

The ultrasound equipment used for the measurements was Voluson e6 by GE Healthcare. The probe type was transvaginal probe RIC5-9W-RS. The measurement procedure consisted in the operator (a medical doctor) indicating the measurement points during the diagnostic procedure with the equipment calculating the distances automatically.

Of course, due to the diverse possible shapes of the endometrium, it would sometimes be possible to measure additional widths (w5,…). However, in the process of building the predictive model, the aforementioned set of parameters was used, as it could be determined for all the analyzed cases.

To determine the parameters describing endometrial compaction, the above measurements were taken at two time points:

- on the day following the ovulatory peak (parameters l1_1, l2_1, w1_1, w2_1, w3_1, and w4_1),
- at the time of embryo transfer into the uterine cavity (parameters l1_2, l2_2, w1_2, w2_2, w3_2, and w4_2).

When determining the extent of variation in the four consecutive measurements of endometrial width (w1–w4), a larger spread in the values of these parameters may indicate that endometrial compaction occurred only in a portion of the endometrial area, e.g., only around the cervical region. The differences and ratios between the smallest and largest widths were calculated. It should be noted that the difference and the ratio do not convey the same information: the difference provides information only the absolute change in the parameter value, while the ratio, on the other hand, refers to the relative change relative to the baseline value:

- max-min = max(w1, w2, w3, w4) − min(w1, w2, w3, w4);
- min_to_max = min(w1, w2, w3, w4)/max(w1, w2, w3, w4).

Thus, the following parameters for the measurements on the day following the ovulatory peak were created:

- max-min_1 and min_to_max_1;

while for the measurements at the time of embryo transfer into the uterine cavity, the following parameters were created:

- max-min_2 and min_to_max_2.

To normalize the obtained measurements according to different uterine lengths (parameter l1), the ratios of the remaining parameters were calculated in relation to the l1 value, resulting in the following ratios:

- l2n_1 = l2_1/l1_1; w1n_1 = w1_1/l1_1; w2n_1 = w2_1/l1_1; w3n_1 = w3_1/l1_1; w4n_1 = w4_1/l1_1 (for the measurements on the day following the ovulatory peak),

and:

- l2n_2 = l2_2/l1_2; w1n_2 = w1_2/l1_2; w2n_2 = w2_2/l1_2; w3n_2 = w3_2/l1_2; w4n_2 = w4_2/l1_2 (for the measurements at the time of embryo transfer into the uterine cavity).

The above parameters refer to the static assessment of the endometrial shape at the specified times. The following ratios of analogous parameters at both time points were also calculated:

- prop_l1 = l1_1/l1_2; prop_l2 = l2_1/l2_2; prop_w1 = w1_1/w1_2; prop_w2 = w2_1/w2_2; prop_w3 = w3_1/w3_2; prop_w4 = w4_1/w4_2,
- prop_l2n = l2n_1/l2n_2; prop_w1n = w1n_1/w1n_2; prop_w2n = w2n_1/w2n_2; prop_w3n = w3n_1/w3n_2; prop_w4n = w4n_1/w4n_2,
- prop_max-min = max-min_1/max-min_2; prop_min_to_max = min_to_max_1/min_to_max_2.

**Figure 1:**
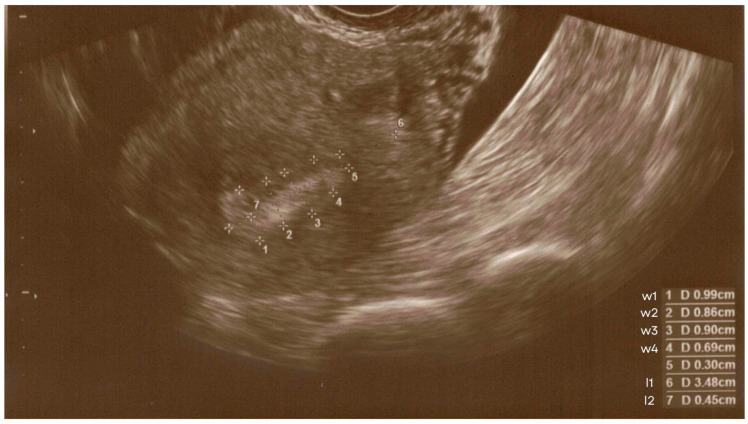
Ultrasound image of the endometrium with measurement sites indicated.

Figure 1 shows an example ultrasound image of the endometrium with the measurement sites described above.

The presence embryo implantation in the endometrium was adopted as the modeled dependent variable. For this variable, a univariate logistic regression analysis was performed, with all the measured endometrial dimensions at both time points, as well as the differences and ratios of the corresponding parameters, used as independent variables. A multivariable logistic regression model was then created. The goodness of fit of the model was assessed using the Hosmer–Lemeshow goodness-of-fit test after logistic modeling. Based on this model, a predictor of the occurrence of implantation was proposed (IMP). An ROC analysis was performed for the created IMP, with the area under the curve (AUC) being determined. Finally, the Mann–Whitney U test was used to compare the values of the IMP between the pregnancy and no pregnancy groups. The IMP values were also divided into four quartile ranges based on the median and quartiles, and the strength of the relationship between these intervals and the percentage of recorded pregnancies in each of them was assessed using Pearson’s Chi-square test of independence.

The Shapiro–Wilk test was used to assess the normality of the distribution of variables. Normality was not observed for the analyzed numerical parameters. Statistically significant results were considered at the level of *p* < 0.05. The Statistica 13.3 package (TIBCO Software Inc., San Ramon, CA, USA) was used for data analysis, while modeling was performed using Stata 18.5 (StataCorp LLC, College Station, TX, USA).

## 3. Results

The basic descriptive statistics for the analyzed parameters, divided into the pregnancy and no pregnancy groups, are presented in Table 1.

None of the listed parameters showed statistically significant differences when compared between the pregnancy and no pregnancy groups. Table 2 presents the results of the univariate logistic regression analysis conducted for all parameters describing endometrial dimensions at both time points, as well as for the calculated ratios, in relation to the dependent variable indicating the occurrence of implantation.

Table 3 presents the created multivariable logistic regression model, based on which the following predictor was proposed:

IMP = 85.34801∙l2n_1 + 42.5543∙w1n_1 + 21.13383∙w2n_1 + 31.95039∙w3n_1 - 89.38451∙w4n_1 − 6.920371∙max-min_1 + 10.13391∙min_to_max_1 - 126.2781∙l2n_2 − 7.97039∙w2n_2 − 4.659379∙prop_w1 − 20.73344∙prop_l2n

**Figure 2:**
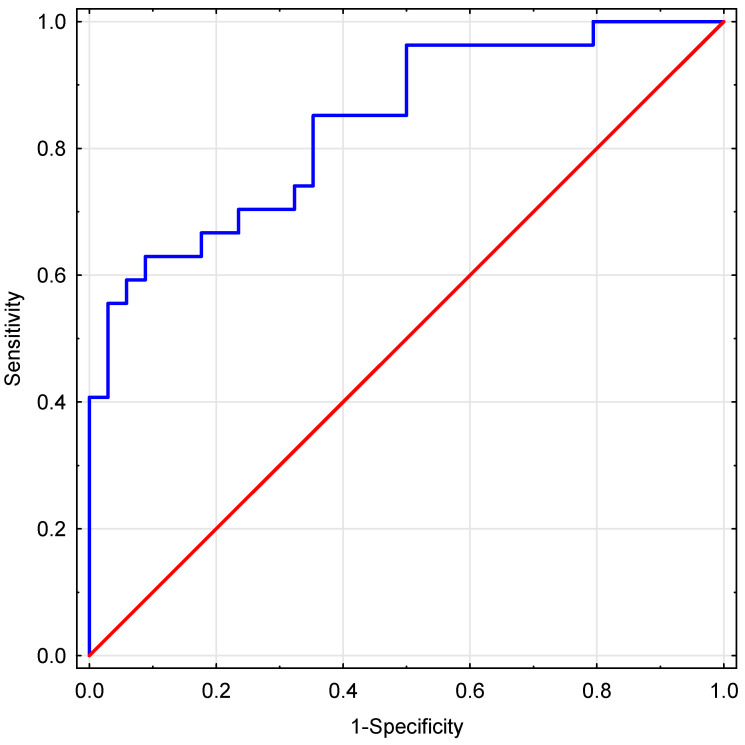
The ROC curve for implantation prediction by the IMP (AUC = 0.839; 95% CI: 0.739–0.938).

A high goodness of fit was confirmed for the model using the Hosmer–Lemeshow goodness-of-fit test after logistic model at *p* = 0.41. The ROC analysis conducted for the proposed IMP yielded an area under the ROC curve (AUC) value of 0.839 (95% CI: 0.739–0.938). The ROC curve is shown in Figure 2.

**Figure 3:**
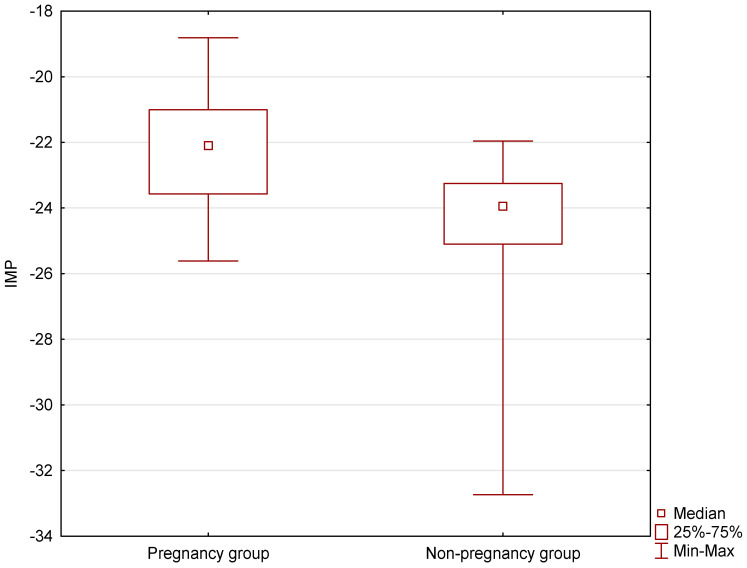
Differences in the IMP (*p* < 0.0001) between groups with and without pregnancy (median, quartiles, and min-max).

Statistically significant differences in the values of the created IMP were observed between the pregnancy and no pregnancy groups at the level of *p* < 0.0001 (Figure 3). The median value of the IMP in the pregnancy group was Me = −22.0972 (Q_1_ = −23.5695; Q_3_ = −21.0082), while in the no pregnancy group it was significantly lower, with Me = −23.9482 (Q_1_ = −25.1003; Q_3_ = −23.2514).

Finally, the values of the IMP were divided into four quartile ranges based on the values of the median and quartiles (Me = −23.3589; Q_1_ = −24.5274; Q_3_ = −22.1521). Statistically significant relationship (*p* < 0.0001) between the individual ranges and the percentage of recorded implantations was found. The obtained percentages of implantation in the different ranges are presented in Table 4. Special attention should be paid to the extreme ranges: in the first range, 15 out of 16 cases did not result in pregnancy, while in the last range, 14 out of 15 cases resulted in pregnancy.

## 4. Discussion

The topic of assessing EC in the context of the endometrium’s ability to accept the ovum—or endometrial receptiveness—was proposed fairly recently by Casper et al., in their studies published in 2019 [15] and 2020 [16], where they assess endometrial thickness at the following two time points: around the ovulatory peak—defined as the end of the estrogen phase—and the day of transfer. The principle is that the shrinkage/compaction of the endometrium between these time points is supposed to suggest better preparation of the endometrium for embryo implantation. In the follicular phase, i.e., before ovulation, estrogen causes the endometrium to grow rapidly as glands and vessels proliferate, and the lining becomes thick. After ovulation, progesterone levels increase, which stops further thickening, but the glands and vessels continue to mature and compact the tissue. In addition, implantation is promoted by the accumulation of glycogen in the endometrium and the appearance of immune cells. These changes are associated with the process of decidualization, during which the stromal cells of the endometrium transform into specialized decidual cells that secrete substances conducive to embryo implantation. At the same time, vascular resistance is reduced, which improves blood supply and facilitates the exchange of substances between the woman’s body and the embryo. As a result of these changes, the endometrium also becomes more homogeneous and dense on ultrasound, even though its actual thickness does not actually increase and may even decrease slightly. Endometrial compaction thus means that the lining has responded well to progesterone stimulation and is ready to accept the embryo. For this reason, the degree of compaction may be used as an indicator of whether the endometrium is receptive and favorable for pregnancy [20,28,29].

Despite the crucial conceptual developments presented above, initiated by Casper et al. [15,16], their studies unfortunately do not present the technical details necessary for understanding and replicating the methodology and thus performing objective and consistent measurements of the thickness of endometrium, e.g., the precise site(s) of measurement, the instruments to be used, or standardized measures. Subsequent studies performed by numerous centers worldwide aimed to confirm or refute the findings reported by Casper et al. [15,16] and they can be quite evenly divided into those that partly confirm the results [17,18,19,20,30,31], and those that disprove the findings or are unable to provide a conclusive answer [21,22,23,24,25,32,33,34,35,36]. However, in light of the aforementioned methodological omissions, it must be emphasized that the problem persists, as none of these studies describe in sufficient detail key aspects such as what exactly is meant by ‘endometrial thickness’, how it is measured, or the exact site of measurement, among other crucial specifics. Consequently, the fact that varied and incompatible approaches to endometrial thickness measurement were used by different teams of researchers can explain the aforementioned discrepancies in the findings of studies attempting to verify the results obtained by Casper et al. [15,16]. In order to obtain consistent results, however, uniform and compatible measurement criteria must be used across different studies, which is the necessary condition for obtaining comparable results that would form the conceptual background of further research.

Furthermore, depending on the site and method of measurement of the value of a parameter, the result may be vastly different and thus may or may not yield meaningful insights into the process. In addition to the methodological inconsistencies outlined above, this strongly implies that performing a single measurement is an insufficient approach, as depending on the exact spot, the endometrium may be characterized by different thickness and undergo various rates of changes [26]. This is why the authors of this study have proposed an approach based on measuring several parameters that describe the endometrium at a given moment, reflecting the studied phenomena much more precisely than a single measurement would. The authors are aware that an approach that requires introducing a larger number of parameters may prove confusing and considerably more difficult to understand as, ideally, only a few variables that explain the whole phenomenon would be preferable. However, as mentioned above, previous studies indicate that as far as endometrial dimensions are concerned, such an approach is clearly insufficient, which is why a slightly more complex methodology is necessary in order to obtain reliable and unambiguous results. Indeed, measuring several parameters at two time points makes it possible to determine the shape of the endometrium and the dynamics of endometrial compaction. Specifically, knowledge about several dimensions of the endometrium allows for estimating the ‘shape’ of the entire organ (e.g., ‘thicker near the uterine body and compacted closer to the cervix’), whereas obtaining the same set of measurements at two time points provides information about the changes the endometrial shape undergoes, which can be described as the ‘dynamics’ of endometrial changes.

The approach described above formed the conceptual basis for the model created in this study. The model contains three groups of parameters:

- Referring to the dimensions and shape of the endometrium on the day following the ovulatory peak;
- Referring to the dimensions and shape of the endometrium at the time of transfer;
- Referring to changes between the two time points (parameters calculated as the ratio of measurements at both time points).

It must be noted that the proposed model is characterized by a very high predictive value (AUC = 0.839), However, in order to responsibly use it for prediction in clinical practice, it must be emphasized that further studies are required to validate it on independent datasets and to test its ‘transferability’ to various clinics, treatment and laboratory procedures and protocols, and other factors specific for a given facility, such as, for instance, varying forms of medications such as progesterone [37,38,39].

The model is based on as many as 11 of the parameters mentioned above, some of which are statistically significant, while others did not reach the established threshold of statistical significance (the highest *p*-value among the parameters included in the multivariable model is *p* = 0.334, while the lowest *p* = 0.012). Obviously, the levels of statistical significance of the individual parameters included in the model do not matter as the focus is only on the predictive value of the constructed IMP. However, feeling that the *p*-values of individual parameters provide additional useful insight, the authors of this study have also attempted to interpret the proposed model with respect to which parameters, and in what direction of change, are the most optimal for implantation. It also needs to be emphasized that, within the study group, the model can reliably identify about one-quarter of cases where implantation is highly likely to occur and another quarter where implantation is highly unlikely. The strong discriminatory power in half of the cases, i.e., the two extreme quarters, implies that the IMP has considerable practical value—after validation and refinement in future studies. Even though accurate prediction for the other half of patients, i.e., the two middle quarters, is not possible in the case of an algorithm based solely on endometrial data—due to the high unpredictability concerning the values of the involved variables—the discriminatory power already achieved in the extreme quarters suggests that the refined version of the IMP may become a useful clinical tool.

In this context, it should also be noted that when simple comparisons—or univariate analyses—were performed, no parameter reached the level of statistical significance, which means that on its own, none of the tested parameters is able to explain the tested phenomena. Hence, if only the aforementioned simple analyses had been taken into consideration, then it would have led to the conclusion that the assessment of endometrial compaction is a useless approach. Indeed, this might have been the case with regard to the studies mentioned earlier, aimed at verifying the findings obtained by Casper et al. [15,16], are concerned [21,22,23,24,25,32,33,34,35,36]. However, the model designed in this study is based on as many as 11 parameters and aims to reflect the dynamics of changes in the shape of endometrium. Although taken individually the parameters do not provide much insight into the tested processes, interestingly, the multivariate approach combines them into a useful model, in which some of them reach the level of statistical significance, while the model itself achieves a high predictive power for implantation.

Finally, the model must be interpreted in the context of those values of its parameters that are conducive to achieving pregnancy, i.e., those that make it possible to achieve endometrial receptiveness. It should be noted that the parameters included in the model reflect only the shape and dimensions of the endometrium and thus cannot be interpreted as pertaining to other aspects such as, for instance, ovum quality.

When explaining certain technical aspects that were taken into account in constructing the IMP, it is worth noting that it is based on the coefficients of individual variables rather than on the odds ratios (OR = *e*^coefficient^) commonly used in the literature. This is because it was created from the formula describing the multivariable logistic regression model, in which the values of individual parameters are multiplied directly by the coefficients. The reason why OR values are not presented in this study is because their interpretation refers to unit changes and thus depends on the units used. In this study, endometrial thickness was expressed in centimeters, whereas the observed changes often concerned differences in millimeters or even fractions of a millimeter. Hence, interpreting the odds ratio for a unit change of 1 cm would yield non-intuitively large values, thus distorting the understanding and interpretation of the obtained results.

The model includes as many as seven parameters referring to the image of the endometrium on the day following the ovulatory peak. These are as follows: the normalized distance from the highest point of the uterine fundus to the first endometrial width (l2n_1); all four normalized widths (w1n_1 to w4n_1), and two parameters referring to the minimum and maximum endometrial widths (max_to_min_1 and min_to_max_1). Positive coefficients of the parameters l2n_1 and the first three widths (w1n_1 to w3n_1) suggest that higher values of these dimensions in the first measurement increase the chances of implantation, whereas the negative coefficient of the fourth width (w4n_1) suggests, on the contrary, that smaller values of this parameter on the day following the ovulatory peak favor implantation. All the above parameters are normalized to the length of the uterus, making them resistant to individual differences in the dimensions of this organ between patients. This leads to the conclusion that an endometrium optimal for embryo implantation should have the largest possible dimensions on the day following the ovulatory peak, but with a distinct narrowing toward the cervix. The significance of this narrowing is confirmed in the literature data [40] as well as through observation of the infrequent cases of patients in whom it is absent or less pronounced, as in these particular cases embryo implantation fails to occur.

The remaining two of the aforementioned parameters (max_to_min_1 and min_to_max_1), which refer to the first measurement on the day following the ovulatory peak, concern the relationship between the minimum and maximum values of the four endometrial width measurements, and indicate that the absolute difference between the largest and smallest width should not be too large. This is also supported by the fact that the relative ratio of minimum to maximum width should optimally have higher values. However, both the relatively small values of the coefficients that correspond to these parameters (−6.9 and 10.1, respectively) and the relatively high *p*-values (0.187 and 0.334, respectively)—indicating no statistically significant association—suggest that their impact on the effectiveness of the proposed model should not be overestimated.

As far as the second time point of endometrium measurement is concerned, i.e., on the day of transfer, the model includes only the following two parameters: the normalized distance from the highest point of the uterine fundus to the first endometrial width (l2n_2) and the second normalized width (w2n_2). It is worth noting that both parameters have negative coefficients and that the former is characterized by the lowest *p*-value (*p* = 0.012)—and thus the highest level of statistical significance—among all the coefficients included in the model. This means that the model identifies benefits of smaller normalized endometrial dimensions on the day of transfer, which confirms the importance of endometrial compaction in the context of subsequent embryo implantation. It may be assumed that Casper et al. [15,16], as well as other researchers who confirmed these results in their studies [17,18,19,20,30,31], managed to observe this very fact. However, as mentioned earlier, it is difficult to verify this insight precisely due to the lack of detailed descriptions of measurement methodology in their studies.

The remaining two parameters measured at the second time point of endometrium assessment concern the ratios of analogous parameters from the first and second measurements, i.e., the first width (prop_w1) and the normalized distance from the highest point of the uterine fundus to the first endometrial width (prop_l2n). Both ratios confirm the aforementioned necessity for these dimensions to decrease between the day following the ovulatory peak and the day of transfer—indicating that the occurrence of endometrial compaction is necessary in order to increase the chances of embryo implantation. It is noteworthy that both parameters have *p*-values in the model below the set threshold of statistical significance (0.04 and 0.029, respectively).

## 5. Conclusions

The obtained results demonstrate the potential to predict pregnancy outcomes based solely on information regarding endometrial receptivity, without taking into account oocyte quality, maternal age, and other factors whose influences on reproductive success have already been established. The created model shows that while an endometrium with optimal receptivity for accepting a developing embryo should be thicker during the ovulatory peak, it should also exhibit narrowing toward the cervix. By the time of transfer, endometrial compaction should occur, resulting in a decrease in endometrial thickness, particularly in the area of the uterine body and in the region where the endometrial width was originally the greatest. Considering this outcome, it is important to emphasize that combining information, in the next step, on endometrial receptivity with data obtained from other sources that describe phenomena known to affect pregnancy outcomes—such as ovum quality or woman’s age—may yield a predictor of even greater power. After refinement, validation, and confirmation of its quality and reliability, it could make it possible to predict infertility treatment outcomes with very high accuracy. Even though at this stage it has been shown to satisfactorily predict approximately half of the cases, its future version—incorporating data other than those concerning endometrial receptivity—is expected to considerably improve on this outcome. This, in turn, would enable clinicians to tailor treatment based on the predicted results, which should lead to a significant improvement in the success of ART-based infertility treatments. This approach and direction will constitute the next planned stage of studies, building on the findings presented in this study.

## 6. Limitations of the Study

The study was performed on a relatively small sample of patients (61). Although it was a sufficient group for the purpose of this research, i.e., testing a novel hypothesis, in order to confirm the results, studies performed in larger groups are necessary. In addition, the study was performed in a single fertility center, which may have resulted in a study group that does not reflect the characteristics of the general population. Although the retrospective character of the study fitted its intended aim, prospective research could potentially have more accurate results.

## Figures and Tables

**Table 1:** Descriptive statistics of parameters included in the analyses.

	Pregnancy					No Pregnancy				
	Min	Q_1_	Me	Q_3_	Max	Min	Q_1_	Me	Q_3_	Max
l1_1	3.150000	3.380000	3.820000	4.620000	5.510000	3.110000	3.660000	4.130000	4.490000	5.12000
l2_1	0.470000	0.620000	0.690000	0.760000	0.940000	0.430000	0.620000	0.750000	0.840000	1.13000
w1_1	0.560000	0.870000	1.080000	1.160000	1.940000	0.660000	0.890000	0.990000	1.200000	1.53000
w2_1	0.560000	0.820000	1.000000	1.120000	1.660000	0.650000	0.810000	0.955000	1.040000	1.68000
w3_1	0.290000	0.670000	0.800000	1.010000	1.170000	0.520000	0.660000	0.820000	0.930000	1.40000
w4_1	0.270000	0.480000	0.610000	0.690000	1.000000	0.230000	0.530000	0.590000	0.760000	1.08000
l2n_1	0.094374	0.133962	0.184275	0.201571	0.246057	0.102381	0.157044	0.180101	0.207407	0.24565
w1n_1	0.143376	0.211382	0.267813	0.328267	0.415254	0.173210	0.210526	0.246579	0.288136	0.40244
w2n_1	0.147005	0.208672	0.257757	0.285714	0.349398	0.154762	0.204918	0.236417	0.276968	0.37805
w3n_1	0.077540	0.190476	0.209719	0.238482	0.259887	0.123810	0.174825	0.195583	0.225058	0.30435
w4n_1	0.077540	0.115090	0.150888	0.179545	0.216450	0.064972	0.123047	0.148948	0.182628	0.23889
max-min_1	0.150000	0.330000	0.480000	0.610000	1.300000	0.070000	0.270000	0.375000	0.600000	0.91000
min_to_max_1	0.243243	0.415730	0.548387	0.669903	0.869565	0.270588	0.504274	0.595733	0.704918	0.92473
l1_2	2.870000	3.260000	3.590000	4.260000	4.990000	2.760000	3.030000	3.450000	3.870000	5.63000
l2_2	0.340000	0.580000	0.640000	0.710000	0.950000	0.350000	0.540000	0.725000	0.810000	0.98000
w1_2	0.620000	1.000000	1.120000	1.380000	1.720000	0.740000	0.840000	1.065000	1.360000	1.68000
w2_2	0.430000	0.770000	1.040000	1.180000	1.570000	0.450000	0.850000	1.035000	1.230000	2.82000
w3_2	0.220000	0.450000	0.640000	0.960000	1.580000	0.270000	0.500000	0.640000	0.860000	1.47000
w4_2	0.140000	0.210000	0.380000	0.540000	1.020000	0.110000	0.220000	0.270000	0.500000	1.14000
l2n_2	0.111579	0.152582	0.182353	0.198630	0.246753	0.108025	0.176259	0.195357	0.223118	0.25887
w1n_2	0.181053	0.261097	0.312684	0.351724	0.473333	0.181818	0.263441	0.303818	0.378897	0.52665
w2n_2	0.137821	0.206316	0.277354	0.328691	0.392330	0.162455	0.235872	0.286883	0.330900	1.00000
w3n_2	0.070513	0.142361	0.185263	0.250653	0.362832	0.089109	0.137592	0.189243	0.247002	0.35185
w4n_2	0.042254	0.065089	0.091922	0.159159	0.238235	0.034161	0.063107	0.084562	0.146199	0.35185
max-min_2	0.170000	0.500000	0.870000	1.050000	1.210000	0.030000	0.520000	0.715000	1.250000	2.64000
min_to_max_2	0.106870	0.179245	0.267606	0.526316	0.817204	0.063830	0.155844	0.288546	0.490196	0.97368
prop_l1	0.772300	1.000000	1.126667	1.200000	1.345133	0.695481	1.050676	1.209555	1.268817	1.56318
prop_l2	0.770115	1.000000	1.061538	1.153846	1.382353	0.767123	0.918033	1.065067	1.200000	2.45714
prop_w1	0.640288	0.823944	0.903226	1.000000	1.580645	0.535714	0.825000	0.952127	1.051020	1.28421
prop_w2	0.584746	0.826531	1.009346	1.223881	2.604651	0.265957	0.789474	0.980672	1.130435	1.90769
prop_w3	0.634146	0.879699	1.216667	1.666667	4.045455	0.537415	0.931034	1.290213	1.656250	2.91667
prop_w4	0.627451	0.888889	1.415094	2.833333	4.357143	0.333333	1.261905	1.936027	3.315789	6.14286
prop_l2n	0.754386	0.862686	0.966652	1.064486	1.494038	0.622726	0.760278	0.931364	1.131368	1.96087
prop_w1n	0.560085	0.703989	0.778867	1.061688	1.518134	0.445033	0.711756	0.836432	0.954678	1.15300
prop_w2n	0.512077	0.723613	0.901099	1.212915	2.214308	0.204918	0.679025	0.801368	0.993731	1.82045
prop_w3n	0.471438	0.796607	1.162652	1.581655	3.439188	0.605765	0.849541	1.091373	1.349872	2.44531
prop_w4n	0.477914	0.922538	1.157804	2.652660	3.704165	0.266010	1.043209	1.609262	2.774903	5.06786
prop_max-min	0.123967	0.395349	0.554348	0.860000	5.529412	0.099237	0.378788	0.561318	0.788462	20.33333
prop_min_to_max	0.441192	1.029160	1.828227	3.174383	4.916388	0.394212	1.293214	2.184134	3.626462	9.32959

**Table 2:** Univariate logistic regression analysis in relation to implantation.

	Coefficient	*p*-Value	95% CI	
l1_1	−0.0755628	0.86	−0.9151631	0.7640375
l2_1	−2.890385	0.165	−6.972129	1.191359
w1_1	0.5492968	0.608	−1.549533	2.648126
w2_1	0.6874133	0.554	−1.58746	2.962287
w3_1	0.1598499	0.909	−2.574738	2.894438
w4_1	−1.366308	0.379	−4.410202	1.677586
l2n_1	−5.335398	0.475	−19.97946	9.308665
w1n_1	3.396003	0.41	−4.681329	11.47333
w2n_1	4.265982	0.383	−5.326791	13.85875
w3n_1	2.087339	0.743	−10.39427	14.56895
w4n_1	−6.478169	0.327	−19.4338	6.47746
max-min_1	1.466228	0.197	−0.7601398	3.692596
min_to_max_1	−2.215221	0.194	−5.556702	1.126261
l1_2	0.3877746	0.354	−0.4322397	1.207789
l2_2	−1.463376	0.417	−4.998472	2.07172
w1_2	0.432877	0.644	−1.401309	2.267063
w2_2	−0.5944935	0.421	−2.04117	0.8521826
w3_2	0.3066585	0.725	−1.403011	2.016328
w4_2	0.2805953	0.787	−1.754466	2.315656
l2n_2	−11.17844	0.107	−24.76405	2.407178
w1n_2	−0.7705128	0.827	−7.693178	6.152152
w2n_2	−3.957189	0.24	−10.56319	2.648815
w3n_2	0.0072987	0.998	−7.450405	7.465002
w4n_2	0.0407817	0.992	−7.907539	7.989102
max-min_2	−0.1883919	0.754	−1.368718	0.9919343
min_to_max_2	−0.0391746	0.973	−2.321871	2.243522
prop_l1	−2.412466	0.128	−5.516381	0.6914485
prop_l2	−0.8518554	0.444	−3.032843	1.329132
prop_w1	−0.322234	0.822	−3.127477	2.483009
prop_w2	0.6842587	0.359	−0.7779167	2.146434
prop_w3	−0.0375737	0.925	−0.8246331	0.7494857
prop_w4	−0.2641165	0.207	−0.6745598	0.1463268
prop_l2n	0.2438047	0.818	−1.830998	2.318608
prop_w1n	1.120142	0.39	−1.433748	3.674031
prop_w2n	1.298968	0.112	−0.3026435	2.90058
prop_w3n	0.3042923	0.481	−0.5428716	1.151456
prop_w4n	−0.1668668	0.468	−0.617847	0.2841134
prop_max-min	−0.0593668	0.622	−0.2952486	0.176515
prop_min_to_max	−0.2144875	0.188	−0.5334927	0.1045177

**Table 3:** Multivariate logistic regression model in relation to implantation.

	Coefficient	*p*-Value	95% CI	
l2n_1	85.34801	0.101	−16.60199	187.298
w1n_1	42.5543	0.08	−5.161251	90.26985
w2n_1	21.13383	0.316	−20.13553	62.40319
w3n_1	31.95039	0.1	−6.065938	69.96672
w4n_1	−89.38451	0.069	−185.8188	7.04982
max-min_1	−6.920371	0.187	−17.19519	3.354449
min_to_max_1	10.13391	0.334	−10.40985	30.67766
l2n_2	−126.2781	0.012	−225.3564	−27.19985
w2n_2	−7.97039	0.189	−19.85397	3.913186
prop_w1	−4.659379	0.04	−9.106193	−0.2125642
prop_l2n	−20.73344	0.029	−39.34986	−2.117016

**Table 4:** Pregnancy rates between quarters of the IMP.

Quarter (N)	C1 (16)	C2 (15)	C3 (15)	C4 (15)
Range	IMP < −24.5274	−24.5274 ≤ IMP < −23.3589	−23.3589 ≤ IMP < −22.1521	−22.1521 ≤ IMP
Pregnancy	1 6.25%	7 46.67%	5 33.33%	14 93.33%
Non-pregnancy	15 93.75%	8 53.33%	10 66.67%	1 6.67%

## Data Availability

The data presented in this study are available on request from the corresponding author.
